# Evaluation of penile erection rigidity in healthy men using virtual touch tissue quantification

**DOI:** 10.2478/v10019-012-0012-4

**Published:** 2012-01-12

**Authors:** Xiaozhi Zheng, Ping Ji, Hongwei Mao, Jing Wu

**Affiliations:** Department of Ultrasound, The Fourth Affiliated Hospital of Nantong University (The First People’s Hospital of Yancheng), 14 Yuehe Road, Yancheng 224006, Jiangsu Province, P.R. China.

**Keywords:** virtual touch tissue quantification, axial and radial rigidity, erection, shear wave velocity

## Abstract

**Background:**

The aim of the study was to describe the shear wave velocity (SWV) values of the penis by virtual touch tissue quantification (VTTQ) and to examine the clinical usefulness of this procedure in evaluation of the rigidity changes in penile erection.

**Patients and methods.:**

VTTQ was performed in 37 healthy volunteers. In the course of erection, SWV values of glans penis, corpus penis and radix penis were quantified and grades of erection were documented. The SWV values at different grades of erection were compared.

**Results:**

The axial and radial SWV values of glans penis, corpus penis and radix penis all significantly decreased from Grade 0 to Grade 4 of erection. At Grade 4, they were less than one-third of that at Grade 0 (axial direction: 0.79 ± 0.13 vs. 2.79 ± 0.32 for glans penis, *P*<0.001; 0.77 ± 0.19 vs. 2.84 ± 0.30 for corpus penis, *P*<0.001 and 0.76 ± 0.15 vs. 2.81 ± 0.34 for radix penis, *P*<0.001; radial direction: 0.82 ± 0.15 vs. 2.83 ± 0.31 for glans penis, *P*<0.001; 0.79 ± 0.18 vs. 2.81 ± 0.27 for corpus penis, *P*<0.001 and 0.81 ± 0.16 vs. 2.82 ± 0.33 for radix penis, *P*<0.001).

**Conclusions:**

VTTQ can provide numerical measurements of penile rigidity and can effectively and sensitively indicate the axial and radial rigidity changes in penile erection, which provide a new approach to assessing the erectile function.

## Introduction

Penile erection rigidity is one of the key factors for successful sexual intercourse, as well as an important index in the diagnosis and treatment of erectile dysfunction (ED). Ideally, the evaluation of ED should include the measurement of axial rigidity and radial rigidity. This requires special devices, such as RigiScan, Digital Inflexion Rigidometer. However, each device has its pros and cons, related to availability, convenience, validity and costs. For example, RigiScan is the most widely utilized device for measuring penile radial rigidity^1^–^5^, but this device does not directly determine axial rigidity.^3^,^6^,^7^ On the contrast, Digital Inflexion Rigidometer is a useful device for measuring penile axial rigidity^3^,^8^,^9^, but it does not directly determine radial rigidity. Complete erection rigidity assessment needs a combinatorial use of RigiScan and Digital Inflexion Rigidometer, which increased the cost and inconvenience. Alternative methods for penile erection rigidity assessment are needed.

Ultrasound is widely used for clinical imaging^10^,^11^, and virtual touch tissue quantification (VTTQ) is a new, promising implementation of the ultrasound acoustic radiation force impulse (ARFI) imaging, which can effectively and objectively detect the tissue rigidity by measuring the shear wave velocity (SWV) values.^12^–^14^ Due to the non-invasive and easily accessible nature of VTTQ, this technology makes it possible to conduct a thoroughly evaluation of erectile rigidity at any portion and any direction in the penis without any discomfort and special preparation. Recently, VTTQ has been used to quantify the rigidity of the liver, kidneys, pancreas, spleen, prostate and breast.^12^–^19^

In our recent study, in order to provide a supplementary approach to assess the penile erection rigidity, we described the normal axial and radial SWV values of the penis by VTTQ and examined the clinical usefulness of this procedure in evaluation of the rigidity changes in penile erection.

## Patients and methods

### Study Population

Our study was approved by the local human research ethics committee and free informed consent was obtained from all the subjects. 37 healthy men with a mean age of 34.6 years (range: from 18 to 63 years) were recruited. All the subjects were evaluated by means of clinical and physical assessment, detailed sexual history, laboratory data (glucose, cholesterol, and triglycerides serum levels), and endocrine assays (testosterone, prolactin, follicular stimulation hormone, and luteinizing hormone), electrocardiogram, radiology, ultrasonographic (US) examinations and computer tomography. The inclusion criteria were: absence of any history of focal or diffuse disease at any of the examined organs. The subjects with risk factors for ED, such as diabetes mellitus, hypertension, ischemic heart disease, neurogenic injury to the spinal cord, and with psychogenic factors were excluded from the study.

## Examination protocol

Two urologists with 10 years of experience in diagnosing and treating ED performed clinical assessments. Erectile response was induced by masturbation one hour after 100 mg sildenafil citrate (Pfizer, New York, NJ, USA) administration, and it was evaluated and judged by the urologist and subject at the same time. Penile erection was categorized into four grades using the following criteria.^20^,^21^ Grade 0 (G0), flaccid; Grade 1(G1), mild tumescence; Grade 2(G2), moderate tumescence but inadequate rigidity for vaginal penetration; Grade 3(G3), full tumescence with moderate rigidity allowing vaginal penetration with some difficulty; and Grade 4(G4), full tumescence and full rigidity allowing vaginal penetration without difficulty.

All US examinations were performed by two radiologists with 15 years of experience in US examinations. A Siemens ACUSON S200 0 US system (Siemens, Germany) equipped with a linear array transducer (9L4) was used in this study. A mechanical index of 1.0 and tissue harmonic imaging of 8 MHz were chosen. VTTQ was performed with the preliminary identification of a target region of interest (ROI) (box with fixed dimension of 6×5mm) on a conventional US image. Then, an acoustic push pulse was transmitted immediately on the right side of the ROI where the SWVs were calculated and expressed with a numerical value (metre/second, m/s) as a result of multiple measurements made for the same spatial location.

**FIGURE 1:**
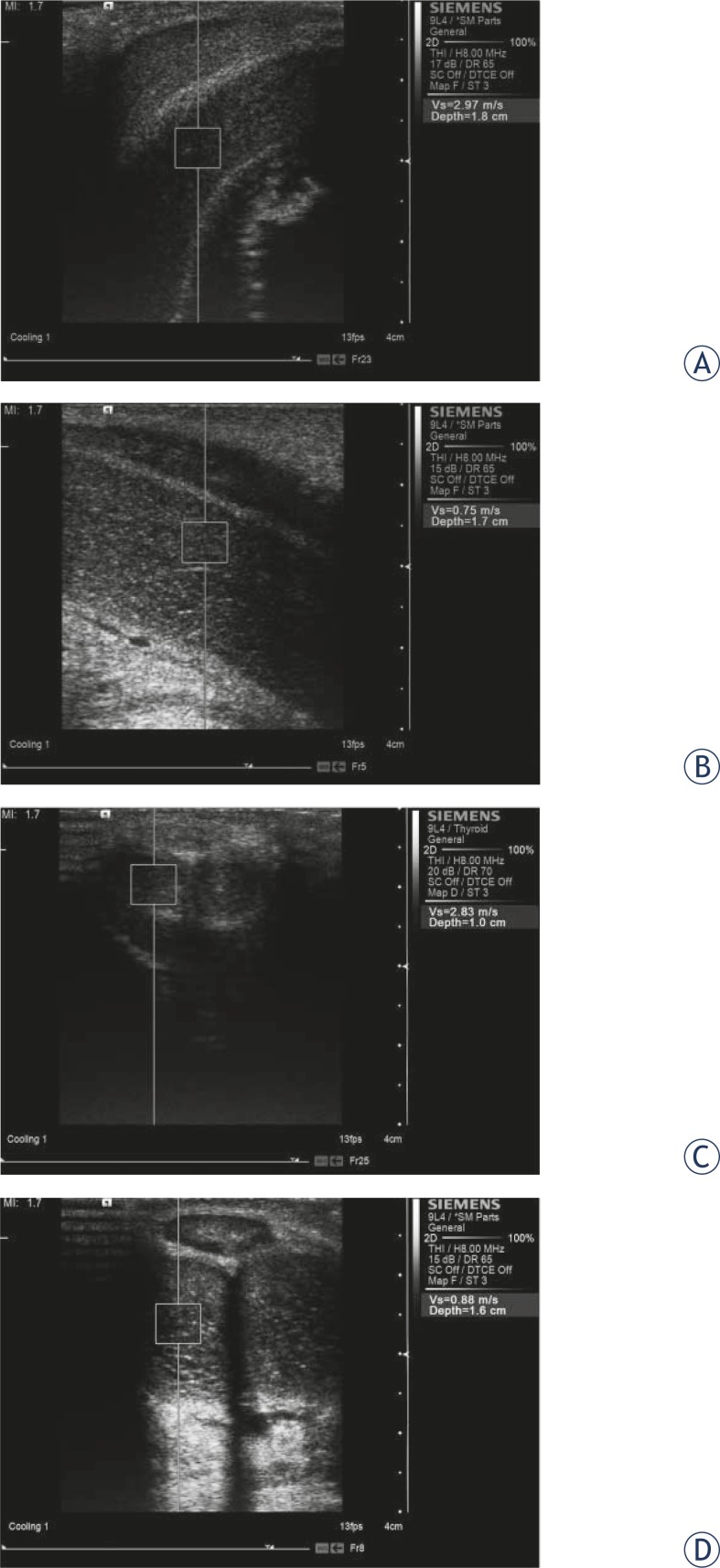
Shear wave velocity measurement in the penis with virtual touch tissue quantification during the erection (A and C: Grade 0; B and D: Grade 4) from the axial direction(A and B) and radial direction (C and D), respectively.

For the penis study, an optional cavernous body was chosen. Firstly, the long axis view was obtained. Three SWV measurements (anterior, center and posterior, *i.e*., axial direction) at glans penis, corpus penis and radix penis in the course of erection were performed, respectively. Secondly, the short axis view was obtained. Three SWV measurements (left, center and right, *i.e*., radial direction) at glans penis, corpus penis and radix penis in the course of erection were performed, respectively (Figure 1). Finally, the average SWV values of each portion were obtained.

## Reproducibility

Intraobserver variability was assessed in 15 subjects by repeating the measurements on two occasions (7 days apart) under the same basal conditions. To test the interobserver variability, the measurements were performed on the same subject by a second observer who was blinded to the first observer’s results. Variability was calculated as the mean percentage error, derived as the difference between the two sets of measurements, divided by the mean observations.

## Statistical analysis and ethical consideration

Data were expressed as the mean ± SD. Differences between the mean values of the two groups were analyzed by unpaired t tests. Differences were considered significant at p<0.05. SPSS version 13.0 (SPSS, Chicago, IL, USA) was used for all statistical analysis.

The study was carried out according to the Declaration of Helsinki.

## Results

**FIGURE 2:**
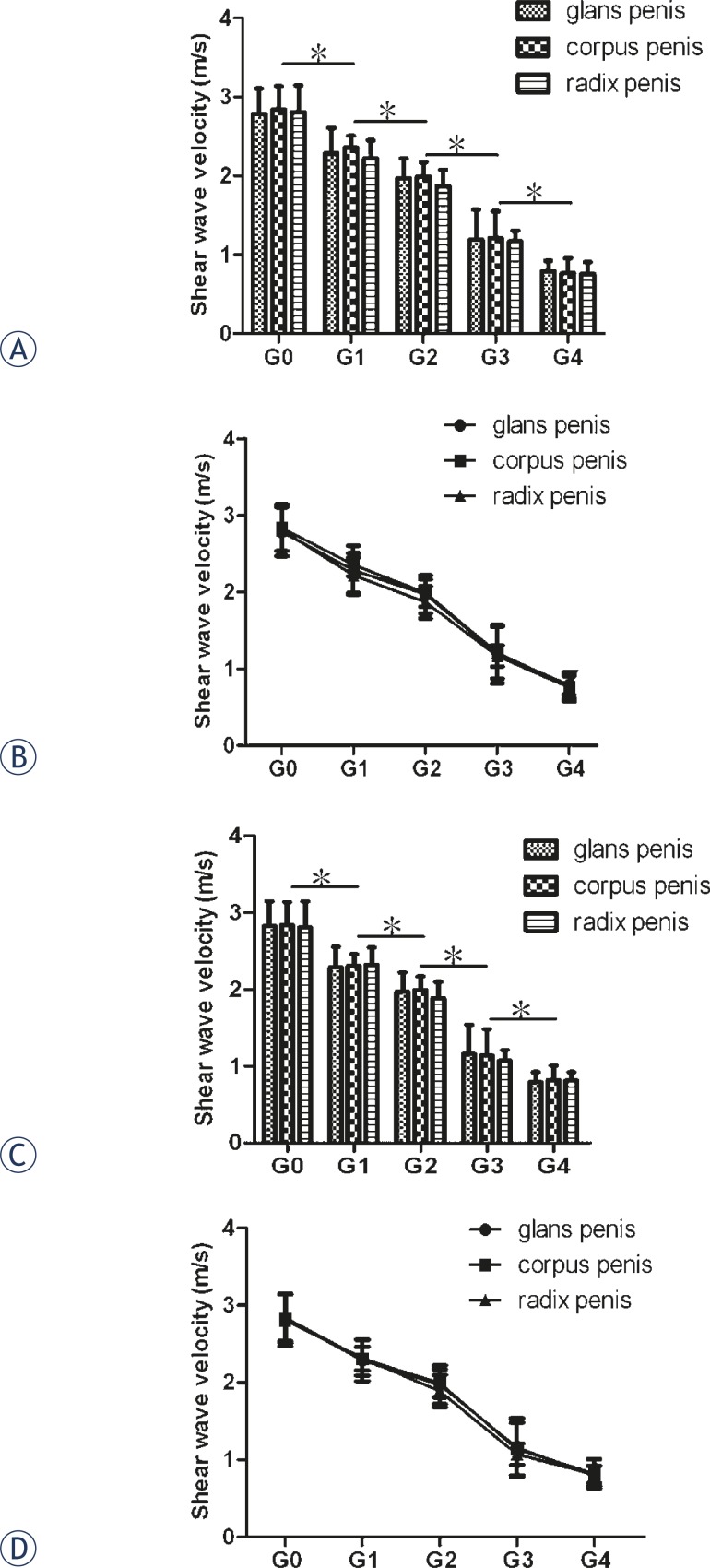
Comparison of penile shear wave velocities among different grades of penile erection. A and B: axial direction; C and D: radial direction. Error bars indicate SD, and asterisks indicate significant differences (*P < 0.05).

As shown in Figure 2, the axial and radial SWV values did not differ at the same portion, neither did they among the glans penis, corpus penis and radix penis at the same grades of erection. They all significantly decreased from Grade 0 to Grade 4 grades of erection. At Grade 4, they were less than one-third of that at Grade 0 (axial direction: 0.79 ± 0.13 vs. 2.79 ± 0.32 for glans penis, *P*<0.001; 0.77 ± 0.19 vs. 2.84 ± 0.30 for corpus penis, *P*<0.001 and 0.76 ± 0.15 vs. 2.81 ± 0.34 for radix penis, *P*<0.001; radial direction: 0.82 ± 0.15 vs. 2.83 ± 0.31 for glans penis, *P*<0.001; 0.79 ± 0.18 vs. 2.81 ± 0.27 for corpus penis, *P*<0.001 and 0.81 ± 0.16 vs. 2.82 ± 0.33 for radix penis, *P*<0.001).

Intraobserver and interobserver variability for shear wave velocity measurements are shown in Table 1. They were all smaller than 5%.

## Discussion

The results presented here indicate that VTTQ can provide numerical measurements of penile rigidity and can effectively and sensitively indicate the rigidity changes in penile erection, which provide a new approach to assessing the erectile function.

ARFI imaging is a new ultrasound imaging modality to evaluate the stiffness of tissues by short-duration acoustic radiation forces that produce localized displacements in a ‘pushed’ ROI,^13^,^22^,^23^ where shear waves (transverses wave propagating perpendicular to the direction of tissue displacement) are generated without the need for external compression.^24^ The induced displacements are indicative of local tissue mechanical properties^14^, and the velocities of shear waves are proportional to tissue rigidity. Fundamentally, SWV is positively correlated with the major mechanical properties indicating material rigidity assuming the material is a linear, isotropic, elastic body.^25^ The stiffer the tissue, the faster the shear wave will be propagated. However, our study showed that the greater the grades of erection, the smaller the SWV values were measured, *i.e*., the stiffer the penis, the slower the shear wave would be propagated. In our study, SWV was negatively correlated with penile rigidity. This finding can be explained by the fact that cavernous body of penis is not a linear, isotropic, elastic body and the penile erection is a multiple neurovascular event as follow^26^: 1) nerve impulses cause the release of neurotransmitters and relaxing factors; 2) relaxation of smooth muscle in the arteries and arterioles supplies the erectile tissue; 3) several times increase in the blood flow to the penis; 4) relaxation of the trabecular smooth muscle increases the compliance of the sinusoids, facilitating rapid filling and expansion of the sinusoidal system; 5) venular subtunical plexes are compressed between the trabeculae and the tunica albuginea, resulting in almost total occlusion of venous out-flow. In the process of penile tumescence, erectile tissue within the ROI gradually decreased and the blood flow within the ROI gradually increased. So the penile SWV values accordingly decreased from Grade 0 to Grade 4 of erection, because a low SWV value, even a “XXXX/0” value is always obtained from the fluids such as blood, water.^13^

In our study, axial rigidity and radial rigidity was separately assessed at the long axis view and the short axis view of the corpora cavernosa by measuring the SWV values. They did not differ at the same portion, neither did they among the glans penis, corpus penis and radix penis at the same grades of erection, because they share a common dependency upon intracavernosal pressure. As a surrogate measure of erection, both axial rigidity and radial rigidity assessed by the SWV values are the most accurate one.

Although VTTQ can potentially be an important quantitative tool for erectile function, there are some limits in the present study. For example, the specimens of subjects are limited. Not all subjects have a satisfactory erectile response to the induction of masturbation after sildenafil citrate administration. Semiquantitative clinical grading of an erection is not very accurate. In the further study, a comparison of VTTQ and RigiScan or Digital Inflexion Rigidometer for the evaluation of penile erection rigidity should be done. There are also some problems with the use of VTTQ for the penile erection rigidity assessment. The fixed box dimension (6×5mm) of the target ROI and the sensitivity to movement artifacts may become obstacles to the extensive application of this new technology.

## Conclusion

This is the first study on the evaluation of penile erection rigidity using VTTQ. Our study indicates that VTTQ can simultaneously provide numerical measurements of penile axial rigidity and radial rigidity at a precise image-based anatomical location, and can effectively and sensitively indicate the rigidity changes in penile erection. Although several limitations mentioned above, this method still holds considerable clinical promise for the assessment of erectile function

## Figures and Tables

**TABLE 1:** Intraobserver and interobserver variability for shear wave velocity measurements.

**Sites**	**Intraobserver variability(%)**	**Interobserver variability(%)**
**Axial direction**		
Glans penis	2.2±1.3	2.6±1.5
Corpus penis	1.9±1.4	1.9±1.7
Radix penis	2.4±1.5	2.3±1.6
**Radial direction**		
Glans penis	2. 3±1. 1	2.5±1.4
Corpus penis	1.9±1.6	1.9±1.5
Radix penis	2.4±1.6	2.6±1.7
